# Status of respectful and non-abusive care during facility-based childbirth in a hospital and health centers in Addis Ababa, Ethiopia

**DOI:** 10.1186/s12978-015-0024-9

**Published:** 2015-04-16

**Authors:** Anteneh Asefa, Delayehu Bekele

**Affiliations:** School of Public and Environmental Health, Hawassa University, Hawassa, Ethiopia; Department of Obstetrics and Gynecology, Saint Paul’s Hospital Millennium Medical College, Addis Ababa, Ethiopia

**Keywords:** Respectful maternity care, Disrespect, Abuse, Childbirth, Quality, Ethiopia

## Abstract

**Background:**

According to the 2011 Ethiopian Demographic and Health Survey, 90.1% of mothers do not deliver in health facilities, with 29.5% citing non-customary service as causative. A low level of skilled attendance at birth is among the leading causes of maternal mortality in low - and middle-income countries.

**Methods:**

A cross-sectional study was undertaken in four health facilities (one specialized teaching hospital and its three catchment health centers) in Addis Ababa, Ethiopia, to quantitatively determine the level and types of disrespect and abuse faced by women during facility-based childbirth, along with their subjective experiences of disrespect and abuse. A questionnaire was administered to 173 mothers immediately prior to discharge from their respective health facility. Reported disrespect and abuse during childbirth was measured under seven categories using 23 performance indicators.

**Results:**

Among multigravida mothers (n = 103), 71.8% had a history of a previous institutional birth and 78% (75.3% in health centers and 81.8% in hospital; p = 0.295) of respondents experienced one or more categories of disrespect and abuse. The violation of the right to information, informed consent, and choice/preference of position during childbirth was reported by all women who gave birth in the hospital and 89.4% of respondents in health centers. Mothers were left without attention during labor in 39.3% of cases (14.1% in health centers and 63.6% in hospital; p < 0.001). Although 78.6% (n = 136) of respondents objectively faced disrespect and abuse, only 22 (16.2%) subjectively experienced disrespect and abuse.

**Conclusions:**

This quantitative study reveals a high level of disrespect and abuse during childbirth that was not perceived as such by the majority of respondents. It is every woman’s right to give birth in woman-centered environment free from disrespect and abuse. Understanding how women define abuse is crucial if Ethiopia is to succeed in increasing the uptake of facility-based births.

## Background

Although there was a substantial decrease in global maternal deaths by 47% between 1990 and 2010, an estimated 287,000 maternal deaths still occurred in 2010, with sub-Saharan Africa (56%) and Southern Asia (29%) accounting for 85% of the global burden. The maternal mortality rate (MMR) in developing regions was 15 times higher than in developed regions: in 2010, sub-Saharan Africa and Ethiopia had the highest MMR with 500 and 676 maternal deaths per 100,000 live births, respectively [1,2]. This high MMR put Ethiopia among seven countries accounting for 3% to 5% of global maternal deaths each.

Maternal mortality is high in countries where the proportion of births attended by skilled providers is low [1]. In Ethiopia in 2010, the proportion of births attended by skilled providers was very low (10%). Poor quality of service, lack of courtesy and respect from providers, fear of exposing the body to strangers, perceived cost of using a health facility, and fear of being attended to by male providers during birth are all known to contribute to low institutional delivery rates [2-5]. In Addis Ababa, the capital city, where the proportion of births occurring in health institutions is high (82.3%, much higher than the national figure), there are growing concerns about the respect and friendliness of safe delivery services.

Respectful and non-abusive care at birth encompasses many points along a continuum spanning dignified, patient-centered care to non-dignified and overtly abusive maternal care. While it is likely that disrespect and abuse are often multi-factorial and may be perceived differently (or even normalized) depending on the specific setting, many stakeholders and maternal health experts agree that disrespect and abuse during facility-based childbirth represent important causes of suffering for women and are important barriers to women choosing to access skilled care [6].

Based on a comprehensive review of the evidence, Bowser and Hill (2010) identified seven categories of disrespect and abuse during childbirth: physical abuse, non-consented care, non-confidential care, non-dignified care, discrimination based on specific patient attributes, abandonment of care, and detention in facilities. It is known, however, that manifestations of disrespect and abuse often fall into more than one category, and these categories are not intended to be mutually exclusive. Rather, categories should be seen as overlapping and representing a continuum [6]. The barriers and facilitators encountered in humanized birth practice can be categorized into four main groups: rules and strategies, physical structure, contingency factors, and individual factors, the most important being the institutional rules and strategies that restrict the presence of a birth companion [7].

Disrespect and abuse of women during childbirth at health facilities have been qualitatively described, but there are little quantitative data. However, there are a limited number of validated quantitative tools that measure satisfaction with care during labor and birth or look specifically at satisfaction at the point of service provision [8]. This study aimed to quantitatively determine the level and types of disrespect and abuse women face during facility-based childbirth and report on subjective experiences of disrespect and abuse.

## Methods and materials

### Study design and setting

A quantitative cross-sectional study using an interviewer-administered questionnaire was conducted to measure the level of disrespect and abuse during facility-based childbirth. The study was carried out in four public health facilities (one specialized teaching hospital and three catchment health centers) in Addis Ababa in August 2013.

### Study population

Women who had given birth vaginally were recruited to the study. Mothers who gave birth via elective or emergency cesarean section were excluded for three reasons: to maintain similarity between the services provided to study subjects between health centers and the hospital; to rule out the effect of anesthesia; and to minimize the time lapse between childbirth and time of interview.

### Sample size and sampling

A single population proportion formula was used to estimate the sample size with assumptions of 5% precision, 95% confidence, and a 10% non-response rate. An assumption that 13% of laboring mothers would face at least one form of disrespect and abuse during childbirth was undertaken. This figure was taken from a previous study conducted in three hospitals in North Ethiopia in which 13% of mothers claimed lack of courtesy and respect from health providers during childbirth services [3]. This proportion was used to obtain a proxy estimate of the sample size required to assess the level of disrespect and abuse, since there has been no prior research in the country that has quantified disrespect and abuse in facility-based childbirth. Therefore, the final calculated sample size was 191. But, 173 mothers who underwent childbirth eventually agreed to participate (response rate, 90.6%). The allocation of the sample to health facilities was made proportionately based on the number of clients who received childbirth services at each facility in the month preceding the data collection period. Thus, 98, 41, 35, and 17 mothers were interviewed from Saint Paul’s Hospital Millennium Medical College (SPHMMC), Kolfe Health Center, Addis Ketema Health Center, and Selam Health Center, respectively. Consecutive interviews were undertaken with mothers for enrollment.

### Data collection

Levels of disrespect and abuse during childbirth were measured using seven performance standards (categories of disrespect and abuse) and their respective verification criteria developed by the Maternal and Child Health Integrated Program (MCHIP) as part of their respectful maternity care tool kit [9]. A total of 23 verification criteria of disrespect and abuse were used in the survey (Table 1). Other pertinent variables (socio-demographic variables, obstetric characteristics, past history of institutional birth, sex of service providers, total length of stay in the health facility, and mothers’ self report of disrespect and abuse during childbirth) were added to the data collection tool as additional information. Since birth companion is not a standard procedure during childbirth in Ethiopia in the public health facility context, the verification criteria enquiring about birth companions was removed. The questionnaire was translated into the national language (Amharic) to ensure clarity of messages and consistency between data collectors. Data were collected immediately prior to discharge from the health facilities after childbirth. Four female data collectors not involved in the women’s care were recruited and trained to use the data collection tool before embarking on data collection.

**Table 1:** **Categories and types of disrespect and abuse reported by mothers during childbirth, Addis Ababa, 2013**

**Categories of disrespect and abuse**	**Types of disrespect and abuse**	**Yes, n (%)**
The woman is protected from physical harm or ill treatment.	• The provider used physical force /slapped me/hit me (n = 171)	4 (2.3%)
	• I was physically restrained (n = 168)	6 (3.5%)
	• I was separated from my baby without medical indication (n = 172)	4 (2.3%)
	• I was denied food or fluid in labor unless medically necessitated (n = 173)	1 (0.6%)
	• I did not receive comfort/pain-relief as necessary (n = 173)	41 (23.7%)
	• The providers did not demonstrate caring in a culturally appropriate way (n = 173)	16 (9.2%)
The woman’s right to information, informed consent, and choice/preferences is protected.	• The provider did not introduce himself/herself to me and my companion (n = 172)	154 (89.0%)
	• The provider did not encourage me to ask questions (n = 172)	109 (63.0%)
	• The provider did not respond to my questions with promptness, politeness, and truthfulness (n = 173)	8 (4.6%)
	• The provider did not explain to me what is being done and what to expect throughout labor and birth (n = 172)	75 (43.4%)
	• The provider did not give me periodic updates on status and progress of my labor (n = 170)	57 (32.9%)
	• The provider did not allow me to move about during labor (n = 172)	35 (20.2%)
	• The provider did not allow to assume position of choice during birth (n = 172)	19 (11.0%)
	• The provider did not obtain my consent or permission prior to any procedure (n = 173)	83 (48.0%)
The woman’s confidentiality and privacy is protected.	• The provider did not use curtains or other visual barriers to protect me (n = 172)	37 (21.4%)
The woman is treated with dignity and respect.	• The provider did not speak to me politely (n = 172)	15 (8.7%)
	• The provider made insults, intimidation, threats, or coerced me (n = 172)	13 (7.5%)
The woman receives equitable care, free of discrimination.	• The provider spoke to me in a language and at a language-level that I cannot understand (n = 171)	33 (19.1%)
	• The provider showed disrespect to me based on any specific attribute (n = 173)	5 (2.9%)
The woman is never left without care/attention.	• The provider did not encourage me to call if needed (n = 172)	51 (29.5%)
	• The provider did not come quickly when I called him/her (n = 173)	8 (4.6%)
	• The provider left me alone or unattended (n = 173)	40 (23.1%)
The woman is never detained or confined against her will.	• I was detained in health facility against my will (n = 173)	1 (0.6%)

### Data quality assurance

Further adjustments to the data collection tool were made after pre-testing it with 3% of the sample size at Shegole Health Center (one of the eight catchment health centers of SPHMMC) to improve clarity, understandability, and simplicity of the messages. Completed questionnaires were checked for completeness and accuracy during the period of data collection.

### Data analysis and interpretation

Data entry, cleaning, and analysis were managed using SPSS version 16 (SPSS, Inc., Chicago, IL) statistical software. Verification criteria were counted within their respective categories of disrespect and abuse. The verification criteria were dichotomized responses, “Yes” or “No”, to objectively identify reported events of disrespect and abuse. For categories of disrespect and abuse with more than one verification criterion, a woman was labeled as “disrespected and abused in the respective category” if she reported “Yes” to at least one of the verification criteria during childbirth. If a mother was identified as having faced disrespect and abuse in at least one of the seven categories, she was considered “disrespected and abused”. Mothers who responded “Yes” to the question “Do you think you have been disrespected and abused during your current childbirth?” were categorized as mothers who experienced disrespect and abuse. Descriptive statistics were used to display the values of the variables in the study; selected variables are presented here. Chi-squared tests were performed to assess statistically significant differences of the level of disrespect and abuse between types of health facilities (health centers and the hospital).

### Ethical considerations

The study proposal received ethical clearance from the Institutional Review Board of SPHMMC. Written permissions were obtained from the health facilities included in the study. Informed written consent was obtained from all clients and health providers who participated in the study after all necessary information were delivered to the study participants including maintenance of anonymity.

## Results

### Socio-demographic and obstetric features of the study participants

Of the total number of women who agreed to participate in the study (n = 173), 42.8% were aged 20–24 years. The mean ± SD of respondents’ age was 25.13 ± 4.30 years. 46.8% of respondents attended primary school and 67.4% were housewives. The majority (82%) of respondents were from Addis Ababa city, and the remaining respondents were from cities neighboring Addis Ababa. During the study, 38.5% of respondents claimed to have an estimated average monthly household income of less than 713 Ethiopian Birr (equivalent to 37.5 USD). The mean gravidity of respondents was 2.1 (+/− SD 1.28), and 39.9% of the mothers were primigravida (Table 2).

**Table 2:** **Socio-demographic and economic characteristics of respondents, Addis Ababa, 2013**

***Characteristics***		***Frequency (%)***
**Age in years**	15-19 years	12 (6.9)
	20-24 years	74 (42.8)
	25-29 years	49 (28.3)
	30 years and above	38 (22.0)
	**Total (N)**	**173 (100.0)**
	**Mean ± SD = 25.13 ± 4.30 years**	
**Religion**	Orthodox	90 (52.0)
	Protestant	21 (12.1)
	Muslim	62 (35.8)
	**Total (N)**	**173 (100.0)**
**Marital status**	Single	11 (6.4)
	Married	160 (92.5)
	Widowed	1 (0.6)
	Divorced	1 (0.6)
	**Total (N)**	**173 (100.0)**
**Educational status**	No formal education	39 (22.5)
	Primary	81 (46.8)
	Secondary and above	53 (30.6)
	**Total (N)**	**173 (100.0)**
**Occupation (n = 172)**	Merchant	9 (5.2)
	Government Employee	6 (3.5)
	House wife	116 (67.4)
	Student	2(1.2)
	Others	39 (22.7)
	**Total (N)**	**172 (100.0)**
**Residential address (n = 172)**	Addis Ababa	141 (82.0)
	Outside Addis Ababa	31 (18.0)
	Total	**172 (100.0)**
Estimated household monthly income in birr (n = 148)*	≤712	57 (38.5)
	≥713	91 (61.5)
	**Total (n)**	**148 (100.0)**
	**Median income = 1000 birr**	

*1USD was equivalent to 19.23 Ethiopian Birr during the study period.

### Respondents’ history of service utilization and experience during their current childbirth in the health facility

Of the interviewed mothers, 95.4% had at least one antenatal care visit during their index pregnancy. From the total number of multigravidas (104), 71.2% had a previous history of an institutional birth. The median duration of stay of respondents in their respective health facility during labor was estimated to be six hours, although 43.9% of mothers stayed for 12 hours or more. Mothers were asked to recall the number of health providers who attended their childbirth. Accordingly, 48% of mothers were attended by more than two service providers at different points during childbirth. The sex of the health provider who mainly attended (as rated by respondents) laboring mothers was reported to be female in 57.8% of scenarios. Moreover, 9% (12.5% in hospital and 5.9% in health centers) of respondents reported that people other than the main service providers had access to see them during childbirth (Table 3).

**Table 3:** **Obstetric and maternal health service use history and experience during current childbirth of respondents, Addis Ababa, 2013**

***Characteristics***		***Frequency (%)***
**Gravidity**	One	69 (39.9)
	Two-three	83 (48.0)
	Four and above	21 (12.1)
	**Total (N)**	**173 (100.0)**
	**Mean ± SD of gravidity = 2.1 ± 1.29**	
**History of ANC use during last pregnancy (n = 170)**	Yes	165 (95.4)
	No	8 (4.6)
	**Total (n)**	**170 (100.0)**
**History of previous institutional birth (n = 104)**	Yes	74 (71.2)
	No	30 (28.8)
	**Total (n)**	**171 (100.0)**
Length of stay in the health facility where childbirth happened* **(n = 171)**	Less than 12 hours	96 (56.1)
	12-24 hours	61 (35.7)
	More than 24 hours	14 (8.2)
	**Total (n)**	**171 (100.0)**
	**Median duration of stay = 6 hours**	
**Number of health professionals who attended the mother at different point in time during childbirth**	One	7 (4.0)
	Two	83 (48.0)
	Three-four	66 (38.2)
	Five and above	17 (9.8)
	**Total (N)**	**173 (100)**
**Sex of the main health provider who attended a mother during childbirth**	Male	73 (42.2)
	Female	100 (57.8)
	**Total (N)**	**173 (100.0)**
**Did anyone other than concerned health provider have access to see you during your labor?**	Yes	16 (9.2)
	No	157 (90.8)
	**Total**	**173 (100.0)**
**Have you faced birth complication/s during your current labor?**	Yes	29 (16.8)
	No	143 (82.7)
	I don’t Know	1 (0.6)
	**Total**	**173 (100.0)**

*total duration of stay from the time of admission until the interview was conducted.

### Categories and types of disrespect and abuse reported by mothers during childbirth

According to the six verification criteria used to identify whether a mother was protected from physical harm or ill treatment during childbirth, 23.7% of respondents claimed that they did not receive necessary comfort/relief measures (Table 1). Furthermore, 2.3% of respondents reported that service providers used physical force or slapped/hit them (Table 1). With respect to the second category of disrespect and abuse (woman’s right to information, informed consent, and choice/preferences protected) that used eight verification criteria, service providers did not introduce themselves to respondents in 89% of cases. Furthermore, periodic updates of labor progress were not given to respondents in 32.9% of cases. On top of this, 43.4% and 48% of respondents reported that providers did not explain what was being done to them and did not obtain their consent or permission prior to any procedure, respectively (Table 1).

Service providers did not use curtains or other visual barriers to protect the mother’s privacy during childbirth in 21.4% of cases (9.5% in health centers and 33% in hospital; p = 0.001). Polite speaking was not used by service providers in 8.7% of childbirths, and 7.5% of respondents reported the occurrence of insults/intimidation or threats/coercion by service providers. In addition, 19.1% of respondents reported the use of unclear or difficult language by providers. Thirty-five percent of mothers in hospital and 10.6% in health centers reported to have been left without attention during the course of labor. Only one respondent (hospital-based) reported that she was detained at the health facility for not being able to pay costs associated with childbirth (Table 1).

### Level of disrespect and abuse reported by respondents

For categories of disrespect and abuse using more than one verification criterion, we counted mothers who faced at least one condition among the possibilities. Accordingly, 32.9% of women were not protected from physical harm or ill treatment during childbirth (27.1% in health centers and 38.6% in hospital; p = 0.105) (Table 4). In addition, the right to information, informed consent, and choice/preference were not protected in nearly 94.8% of respondents (100% in the hospital and 89.4% in health centers; p = 0.002). Specifically, 48% of women were not asked for their consent or permission prior to any procedure. There was a significant difference in not maintaining women’s privacy during childbirth between the surveyed hospital (33%) and the health centers (9.4%; p < 0.0001; Table 4). Similarly, leaving mothers in labor without attention was reported in 39.3% of cases (14.1% in health centers and 63.6% in hospital; p < 0.0001). In order to compute the overall prevalence of disrespect and abuse during childbirth, all 23 verification criteria were checked, and 136 mothers faced at least one form of disrespect and abuse; the overall prevalence of disrespect and abuse during childbirth was, therefore, 78.6% (75.3% in health centers and 81.8% in hospital; p = 0.295; Table 4).

**Table 4:** **Prevalence of disrespect and abuse during childbirth by categories, Addis Ababa, 2013**

**Categories of disrespect and abuse**	**Prevalence in health centers**	**Prevalence in hospital**	**P value**	**Total prevalence**
The woman is not protected from physical harm or ill treatment	27.1%	38.6%	0.105	32.9%
The woman’s right to information, informed consent, and choice/preferences is not protected	89.4%	100.0 %	0.002	94.8%
The woman’s confidentiality and privacy is not protected	9.4%	33.0 %	<0.000	21.4%
The woman is not treated with dignity and respect	9.4%	14.8%	0.280	12.1%
The woman did not receive equitable care, free of discrimination	10.6%	28.4%	0.003	19.7%
The woman is left without care/attention	14.1%	63.6%	<0.000	39.3%
The woman is detained or confined against her will	1.2%	0	0.308	0.6%
Overall prevalence of disrespect and abuse*	**75.3%**	**81.8%**	**0.295**	**78.6%**

*Mothers who faced disrespect and abuse in at least one among the seven categories.

Among respondents who were identified to have faced disrespect and abuse (n = 136), only 22 (16.2%) reported that they had experienced disrespect and abuse. After stratification of disrespect and abuse by socio-demographic and obstetric variables, only respondents’ monthly income was significantly associated with a different level of disrespect and abuse (89.5% among those with a monthly income of <713 birr and 70.3% among those with monthly income of ≥ 713 birr; p = 0.006).

## Discussion

This study investigated the types and levels of disrespect and abuse faced by women during childbirth in different institutional settings using quantitative methods. Over three quarters of women interviewed were identified as having faced at least one form of disrespect and abuse during childbirth. However, only 22 (16.2%) reported that they had experienced disrespect and abuse. In this study, the chance for recall bias was lessened since mothers were interviewed immediately before discharge from the health facility where they had childbirth. Furthermore, quantification of the different types of disrespect and abuse using standard performance indicators addressed the gap (determining the magnitude of disrespect and abuse) which various qualitative studies lack. The study’s limitations include: exhaustively addressing all types of disrespect and abuse that might have been practiced but not captured; delineating urban–rural differences, since the set-up may vary in these different contexts; and non-random selection of mothers, as interviews were conducted on a consecutive basis.

Violating women’s right to information, informed consent, and choice/preferences was the most (94.8%) prevalent component of disrespect and abuse in the studied health facilities. More specifically, almost half of the women did not give their informed consent before any procedure performed in the course of labor. This is contrary to recommendations that state “if interventions become necessary for valid indications, service providers must make the mother aware of the necessity as well as the risks of the intervention so that she can give informed consent” [10]. Health administrators, professionals, and women perceive the humanization of birth as care that includes dignity and respect and that also considers a woman’s right to choose and participate in the decision-making process [11,12]. This evidence clearly underlines the need for greater action towards meeting clients’ expectations; this is emphasized in the customer value proposition of the fourth Ethiopian health sector development programme [13].

According to a systematic review on the importance of continuous support during childbirth, continuous support has clinically meaningful benefit for the health of a woman and her newborn [14]. However, our study demonstrates that 40% of women were left without attention during the course of childbirth, which might negatively contribute to the health of the mother and her newborn. According to a study conducted in Canada, nurses’ intentions to provide continuous labor support to women with and without epidural analgesia were related to their attitudes and subjective norms [15].

With respect to issues related to privacy, 21.4% of respondents reported that service providers didn’t use curtains or other visual barriers to protect them during childbirth. Attendance of unknown and unwanted persons during childbirth has been reported to be associated with dissatisfaction in women using childbirth services in Jordan [16]. The problem of maintaining privacy in the studied health facilities may be due to presence of a large number of health care professionals and student interns who might have interfered with women’s privacy, especially in the hospital; this may instill a lack of intimacy and affect the continuity of care women receive from health institutions [17,18].

Only one study respondent was detained in a health facility for not being able to pay costs associated with childbirth. Perceptions that this is pervasive might cause other mothers to refrain from giving birth in public health institutions and act as a barrier to the effective utilization of public maternity services [19].

In public health facilities in Ethiopia, a woman is not allowed to choose a birth companion for support during the course of childbirth in a labor ward. Denying women this support is not only against evidence-based practice [20], but is also associated with dissatisfaction of women with childbirth services [21]. In addition, institutional rules and strategies that restrict the presence of a birth companion are reported to be a significant barrier to humanized birth care [7,18], exacerbating facility-based disrespect and abuse during childbirth.

Ethiopian women and their families value the supportive and comfortable care they obtain from traditional birth attendants when they give birth at home [22]. This further prevents women from choosing to give birth at health facilities if they are not served respectfully in these settings. Studies from Cambodia and Tanzania have shown that women’s choice of health facility was influenced by their perceptions of safety and staff attitudes [19,23].

The majority of women identified as having faced disrespect and abuse during childbirth actually reported that they were not disrespected and abused, demonstrating that acts that are considered to disrespectful and abusive are not usually considered to be serious by service users. This normalization of disrespect and abuse is a known individual-level contributor to disrespect and abuse during childbirth [6]. This discrepancy between reported and experienced abuse merits closer examination of two factors: 1) whether the subjective experience of disrespect and abuse can be accurately measured quantitatively, and 2) how women experience disrespect and abuse during facility-based care.

Although the total prevalence of disrespect and abuse during childbirth was not significantly different between the hospital and the three health centers included in this study, there were statistically significant differences in the levels of disrespect and abuse in four out of seven categories of disrespect and abuse. A Japanese study showed that women who gave birth at birth centers rated woman-centered care highly and were satisfied with the care they received compared to those who gave birth in clinics and hospitals [24].

The high level of disrespect and abuse during childbirth reported here might have negative consequences for service utilization, since disrespectful and abusive treatment of women during childbirth is a deterrent to the utilization of skilled birth and is in violation of human rights according to various literature [6,25,26]. Moreover, there is documented evidence that supportive behavior during childbirth positively affects birth outcomes. Hence, disrespectful and abusive care during childbirth may negatively affect birth outcomes [27-29].

Generally, women perceive the quality of maternal health services in terms of privacy, adequacy of information, openness to patients, compassion for patients, respect for patients, and time devoted to patients [30]. As such, we believe that by leaving these matters unaddressed, challenges of achieving MDG 5in Ethiopia will persist. In light of this and in line with a systematic review performed to develop a framework for quality maternal and newborn care, health systems should respond to the need to acknowledge and respect the values of clients to improve quality [31].

This study used a quantitative approach to measure disrespect and abuse during childbirth based on indicators that are designed for ongoing quality improvement efforts; however, this may be limited in providing details about disrespect and abuse practices. Although the prevalence of disrespect and abuse is considered difficult to measure due to lack of rigorous definition [25,32,33], this study deployed a recently developed performance indicator to quantitatively describe the prevalence of disrespect and abuse. Facility exit interviews are likely to be the most efficient and least costly way to obtain data from a large sample. Gathering information immediately following delivery reduces the potential for recall bias but does not allow for an understanding of how time and situational circumstances may affect women’s perceptions of disrespect and abuse, which may be critical to their subsequent utilization decisions [26].

The findings of this study alert all stakeholders which aim at reducing maternal mortality by attracting more women to health facility through promotion of customer friendly and respectful maternity care. To this effect it would be very appropriate if the performance indicators used in this study are communicated to health care workers and clients to promote respectfulness of care and to mitigate facility based disrespect and abuse. Future research could adopt a mixed method approach to deliver more complete information on the exact extent (depth) of disrespect and abuse. We also recommend that health administrators and service providers promote and institutionalize locally contextualized respectful maternity care standards to protect women’s rights and to attract women to health facilities.

## Conclusions

Here, we show that quantitative approaches can be used to measure disrespect and abuse during childbirth, although there are indications that quantitative data alone may not be able to capture the nuances and subjective nature of the disrespect and abuse experienced by new mothers. The level of disrespect and abuse during childbirth was very high, and there were significant differences with respect to mothers’ reports of categories of disrespect and abuse between hospital and health centers. It is every woman’s right to give birth in a woman-centered context free from disrespect and abuse; understanding how women define this is crucial if Ethiopia is to succeed in increasing the uptake of high-quality facility-based births. Hence, provision of woman-centered care in a respectful and non-abusive manner needs to be given adequate emphasis to attract more women to health facilities, to make services more woman friendly, and to humanize services.
